# Federated learning framework for IoT intrusion detection using tab transformer and nature-inspired hyperparameter optimization

**DOI:** 10.3389/fdata.2025.1526480

**Published:** 2025-05-14

**Authors:** Mohamed Abd Elaziz, Ibrahim A. Fares, Abdelghani Dahou, Mansour Shrahili

**Affiliations:** ^1^Department of Mathematics, Faculty of Science, Zagazig University, Zagazig, Egypt; ^2^Faculty of Computer Science and Engineering, Galala University, Suez, Egypt; ^3^Artificial Intelligence Research Center (AIRC), College of Engineering and Information Technology, Ajman University, Ajman, United Arab Emirates; ^4^School of Computer Science and Technology, Zhejiang Normal University, Jinhua, China; ^5^Department of Statistics and Operations Research, College of Science, King Saud University, Riyadh, Saudi Arabia

**Keywords:** federated learning, transformers, intrusion detection system (IDS), cybersecurity, Internet of Things (IoT), optimization

## Abstract

Intrusion detection has been of prime concern in the Internet of Things (IoT) environment due to the rapid increase in cyber threats. Majority of traditional intrusion detection systems (IDSs) rely on centralized models, raising significant privacy concerns. Federated learning (FL) offers a decentralized alternative; however, many existing FL-based IDS frameworks suffer from poor performance due to suboptimal model architectures and ineffective hyperparameter selection. To address these challenges, this paper introduces a novel trust-centric FL framework based on the tab transformer (TTF) model for IDS. We enhance the Tab model through an optimization process, utilizing a hyperparameter tuning algorithm inspired by the nature-based electric eel foraging optimization (EEFO) algorithm. The goal of the developed framework is to improve the detection of IDS without using centralized data to preserve privacy. Whereas it enhances the processing and detection capability of huge amounts of data generated from IoT devices. Our framework is tested on three IoT datasets: N-BaIoT, UNSW-NB15, and CICIoT2023 to ensure the model's performance. Experimental results show that the proposed framework significantly exceeds traditional methods in terms of accuracy, precision, and recall. The results presented in this study confirm the effectiveness and superior performance of the proposed FL-based IDS framework.

## 1 Introduction

The Internet of Things (IoT) has evolved an essential component of our daily lives. It enables a wide range of applications, spanning smart homes, healthcare, agriculture, and industrial automation (Sarker et al., 2023). The expansion of IoT devices has also spread new security threats. Intrusion detection systems (IDSs) are necessary for identifying and preventing these threats (Saba et al., 2022). Traditionally, many IDS rely on centralized models, which require data to be transferred to a central server for processing (Dong and Wang, 2016). This approach raises significant privacy concerns, and the dynamic nature of IoT devices further complicates intrusion detection (ID). For instance, these challenges include processing diverse data types and adapting to evolving behavioral patterns. Therefore, there is a need for more advanced IDSs that could effectively address these challenges.

Machine learning (ML) models are employed in various solutions for dynamic and adaptive IDS in IoT (Alsahli et al., 2021; Amiri-Zarandi et al., 2020). These ML-based solutions proved their effectiveness in accurately detecting IoT intrusions. However, concerns have grown regarding data privacy during the learning or training phase. Many current IDS approaches that utilize ML are centralized in nature. These IDS often require large volumes of data, including sensitive and confidential information collected from various IoT devices, to be transferred to central servers. Such an approach exposes the transmitted data to significant privacy risks. Consequently, this may result in data security issues and privacy hazards, potentially discouraging data sharing in such systems (Rahman et al., 2020). Another limitation of centralized IDS is the high computational cost of processing large volumes of data on central servers. Thus, federated learning (FL)-based IDS frameworks could offer viable solutions to these challenges.

The FL is an ML technique that authorizes data to be processed locally at the device level, preserving privacy and reducing communication costs (Li L. et al., 2020). It eliminates the need for data aggregation at a central server by distributing learning operations across multiple participants (Koněcnŷ et al., 2016). In FL-based IDS framework setups, the data remains confidential on local servers, and a replica of the model (learned model) is trained on this local data, where the learned model's weights are transmitted to a central server model (Mabrouk et al., 2023). Such methods reduce the workload on the central server while ensuring data privacy and security (Bonawitz et al., 2019). FL-based IDS frameworks are well aligned with the distributed nature of IoT environments. The edge servers of IoT reserve sufficient computational resources to process tasks at the edge (Li et al., 2018).

The FL-based IDS frameworks offer several benefits for IoT applications. First, it improves data privacy since it treats each IoT device as an individual client and trains models without transmitting its data (Aledhari et al., 2020). Second, FL-based IDS frameworks may increase the scalability of the detection process and cost-efficiency since they authorize simultaneous training of models on datasets kept in multiple locations or servers. Finally, FL-based IDS frameworks enhance data accuracy and diversity, which are critical in data science, as larger and more diverse datasets typically lead to improved model performance. Therefore, FL-based IDS frameworks are considered highly compatible with the distributed nature of IoT devices, particularly when IoT infrastructures leverage edge servers (Nguyen et al., 2021). Recent studies and research have applied the FL-based IDS frameworks approach for the ID in IoT devices (Gouissem et al., 2023). However, these approaches often rely on classical ML models and general hyperparameter optimization techniques, which may not fully exploit the potential of FL-based IDS frameworks (Zhang H. et al., 2023). Recently, integrating DL with FL-based IDS frameworks has emerged as a comprehensive and robust approach to intrusion detection. This integration enables the utilization of diverse IDS types and advanced DL techniques while addressing associated challenges (Agrawal et al., 2022).

Rey et al. (2022) proposed a security framework that utilizes the FL procedure to detect and identify malware affecting IoT infrastructure in a privacy-preserving manner. This framework integrates anomaly detection and classification using multi-layer perceptron (MLP) and autoencoder neural network methods. The authors illustrate a Beyond 5G (B5G) scenario where detecting cyberattacks targeting IoT devices, managing sensitive data (including non-independent and identically distributed [non-IID] data), and addressing untrusted stakeholders or clients are critical. The authors also highlighted additional challenges when implementing traditional ML pipelines in a federated setting, such as normalization, hyperparameter selection, and threshold determination. The limitations of this framework include the following:

- The limited number of clients used in their experiments may not accurately represent real-world scenarios involving millions of devices.
- Communication overhead can be significant, particularly when scaling to a large number of clients.
- The centralized aspects of the framework may raise concerns in privacy-sensitive systems.
- Finally, optimizing hyperparameters in FL-based IDS frameworks remains challenging, particularly when dealing with non-IID data.

To address the aforementioned limitations, this study proposes an improved FL-based IDS framework designed to enhance the detection of intrusions in IoT environments. The core of this enhanced framework lies in the integration of the tab transformer (TTF) (Huang et al., 2020) into the existing framework (Rey et al., 2022). The TTF model replaces classical neural network and autoencoder models, offering superior capability in capturing complex patterns within datasets. Additionally, this study employs the electric eel foraging optimization (EEFO) algorithm for hyperparameter tuning within the enhanced framework (Zhao W. et al., 2023). The EEFO Algorithm, inspired by the foraging behavior of electric eels, efficiently navigates the hyperparameter space. This choice is motivated by the need to optimize the model's performance without incurring the computational expense normally associated with normal methods such as the grid search algorithm. The main contributions of this study are listed in the subsequent points:

- An enhanced federated learning (FL)-based IDS framework that leverages FL to detect intrusions on IoT devices while ensuring data privacy. The proposed framework integrates both anomaly detection and classification methodologies.
- Implementation of the tab transformer (TTF) model, which excels in efficiently handling and processing large-scale IoT data.
- Utilization of the electric eel foraging optimization (EEFO) Algorithm for hyperparameter optimization, serving as a more efficient alternative to the computationally expensive grid search method.
- Comprehensive evaluation of the proposed FL framework by testing it on three distinct datasets: N-BaIoT, UNSW-NB15, and CICIoT2023.

The structure of this paper is systematically organized as follows: Section 2 provides an overview of related studies. Section 3 explores the Preliminaries, offering a foundational understanding of the TTF, EEFO algorithm, and the FL-based IDS framework, all of which are essential for the subsequent discussion. Section 4, introduces the proposed model based on the EEFO algorithm, outlining its design and potential for enhancing IoT cybersecurity. Section 5 describes the experimental setup, elaborates on the dataset details and evaluation metrics, and establishes the groundwork for thoroughly evaluating the proposed framework. Additionally, it presents the experimental results, which provide a critical assessment of the proposed model, along with an analysis of its performance under various adversarial scenarios. The paper concludes with Section 6, which discusses the key findings, implications of the current study, and potential directions for future research.

## 2 Related studies

This Section presents an overview of FL-based IDS frameworks applied recently on IDS systems for IoT environments and underlines their application in IDS. McMahan et al. (2017) presented federated learning (FedAvg), which is an evolving technique for training statistical models on end devices. It concerns data privacy and the escalating computational capabilities of edge devices. FL exemplifies the broader concept of “bringing code to data rather than data to code”. Since its inception, the FL has seen substantial advancements by multiple methodologies designed to address its challenges. Meanwhile, data heterogeneity remains an influential issue within FL approaches. In response, Li T. et al. (2020) presented a modified (FedProx) iteration of FedAvg characterized by the addition of the regularization term to the local objective functions. FedProx aims to relieve inconsistencies arising from training global models on heterogeneous data. Empirical findings indicate that FedProx displays enhanced stability and accuracy within heterogeneous networks compared to FedAvg.

The FL-based IDS frameworks have evolved significantly, as demonstrated by Yang et al. (2019) and Kairouz et al. (2021), which comprehensively explore recent advancements in this field. Because of its decentralized structure, FL distributes risks among diverse entities, especially clients and servers. In Lyu et al. (2022), considerable challenges are highlighted within an adversarial setup along with established defense mechanisms to safeguard the system against these threats. The authors of Biggio et al. (2012) proposed using support vector machines (SVM) to counter various data poisoning attacks.These attacks include label flipping, where the binary labels of specific data points in the training dataset are altered to disrupt the model training process. In Blanchard et al. (2017), the authors investigated the strength of a dispersed accomplishment of Stochastic Gradient Descent when faced with randomly acting (Byzantine) competitors. The study introduced a model strategy for a poisoning attack executed from the perspective of a hostile client adept at estimating gradients. First, it illustrates that the standard model aggregation stage performed by a server in general of FL-based IDS frameworks fails to manage a single hostile client within the FL approach. Furthermore, it establishes a broader demand that no model aggregation function be used, which may rely on linear combinations of client-sent models and could effectively withstand attacks from Byzantine adversaries.

Applying the same hyperparameters across all clients may be ineffective due to differences in dataset volumes and distributions. To optimize metrics acquired during training on each client, client-specific hyperparameter optimization can facilitate the use of hyperparameters; hence, it can reduce the overall convergence duration. Several studies, including FL (Reddi et al., 2020; Rey et al., 2022), have used grid search (Bergstra et al., 2011) to fine-tune the operation for the learning rate parameter on the central server before beginning its training. Nevertheless, this method fine-tunes the learning rate using placeholder datasets, which may not accurately describe the real dataset and therefore become impractical in FL scenarios. In Dai et al. (2020), a new approach that incorporates Bayesian optimization with Thompson selection for optimizing hyperparameters on numerous clients is introduced. FedEx (Khodak et al., 2021), which borrows weight-sharing neural architecture search techniques, presents an FL-HPO framework utilizing the Successive Halving Algorithm (SHA) to expedite generic hyperparameter tuning. However, FedEx also brings an added hyperparameter tuning effect that needs prior tuning. Zhou et al. (2021) implements a one-shot task with Bayesian optimization pre-FL training at clients to derive optimal hyperparameters, later shared with the server for selection and training the final federated model. HANF (Seng et al., 2022) treats the selection of ideal hyperparameters as an n-armed bandit problem and uses weight sharing to find optimal hyperparameters. However, the search strategy for optimal hyperparameters demands large resources of time and capital, especially as the hyperparameter space expands. Although the majority of studies focused on improving the FL-based IDS framework's performance from the server side, none addressed possible improvements at the client end. The majority of the hyperparameter optimization methods demand extra aids to acquire optimal parameters and optimize them individually before implied training (Kundroo and Kim, 2023).

Javeed et al. (2024) presented a novel FL-based IDS for IoT networks that combines FL with a zero-trust security model. The proposed approach employs a hybrid CNN–BiLSTM architecture to extract spatial and temporal features from network traffic, enabling effective detection of diverse cyberattacks while preserving data privacy through local model training and secure weight aggregation. Experimental evaluations on the CICIDS2017 and Edge-IIoTset datasets demonstrate enhanced accuracy and scalability compared to traditional centralized methods. Bukhari et al. (2024) presented a privacy-preserving IDS for wireless sensor networks that leverages FL to collaboratively train a DL model using sensor nodes without exposing local data. They proposed the FL-SCNN-Bi-LSTM model, which combines a Stacked CNN for spatial feature extraction with a Bidirectional LSTM network for capturing temporal dependencies, enabling robust detection of intrusions such as DoS attacks.

Table 1 summarizes recent related studies published in 2023 and 2024, highlighting their advantages and disadvantages. To the best of our knowledge, the proposed framework leverages the TTF as a detection model and employs the EEFO algorithm for adaptive hyperparameter optimization during the training process. This approach harnesses the power of the attention mechanism and fine-tunes hyperparameters on each client to enhance local convergence, thereby improving global convergence at the server.

**Table 1:** Related studies summary.

**References**	**Model used**	**Advantages**	**Disadvantages**
**Zhao R. et al. (2023)**	**Semi-supervised FL via knowledge distillation**	**Better detection performance, lower communication overhead**	**Non-IID data affects training**
Merzouk et al. (2023)	Data poisoning attack parameters	Guidelines for security evaluation	Lack of transparency in federated learning
Sebastian (2023)	SMOTE, outlier detection, hyperparameter tuning	High ID performance, protects sensitive data	Implementation complexity
Novikova and Golubev (2023)	Federated learning-based IDS architecture	Expands variety of data used, increasing detection rate	Requires significant computational resources
Xu (2023)	DPFL-F2IDS	High F1-scores, preserves privacy	Trade-off between utility and privacy metrics
Ruzafa-Alcázar et al. (2023)	FL with differential privacy	Similar results with noise in training	Challenges with privacy concerns in data sharing
Li et al. (2023)	Dynamic weighted aggregation federated learning (DAFL)	Excellent detection performance with low communication overhead	Complex implementation
Lin et al. (2023)	Federated transfer learning	Addresses Non-IID data issues	Varying class outputs challenge
Huang et al. (2023)	Personalized FL Execution & Evaluation Dual Network	Improved model stability, reduces negative influence of FL	High complexity in model personalization
Zhang Q. et al. (2023)	Personalized FL Algorithms	High performance in attack detection under various data distributions	Challenges in handling data heterogeneity
Hao et al. (2024)	FL for hybrid attacks	General architecture enhances client-side defenses	Complexity of integrating multiple defenses
Al Essa and Bhaya (2024)	Hybrid FS	Improved feature selection and classifier performance	Potential scalability issues
Seyed et al. (2024)	Cybersecurity mechanism for IDS	Effective cyber security mechanism for IoT	Requires extensive data for optimal performance
Wang et al. (2024)	LDS-FL	Privacy preservation with high accuracy	Balancing trade-offs between privacy and model utility
Chen et al. (2024)	DFL	Reduces bandwidth consumption with intermediate results	Implementation complexity in real-world scenarios
Javeed et al. (2024)	CNN–BiLSTM-based FL	High detection accuracy, robust performance	High communication overhead and scalability trade-offs with a growing number of edge devices
Bukhari et al. (2024)	FL-SCNN-Bi-LSTM	Privacy preservation, high detection accuracy, effective extraction of spatial and temporal features	Increased communication overhead, implementation complexity, challenges in real-time.
Olanrewaju-George and Pranggono (2025)	AutoEncoder- and DNN-based FL	Data privacy, robust detection, high accuracy	Scalability issues, managing heterogeneous IoT devices, increased communication overhead.
Danquah et al. (2025)	FL-based MLP with XGBoost	High detection performance, low computational complexity, and incorporates differential privacy	limited to four FL clients, scalability challenges, and intensive hyperparameter tuning
Wen et al. (2025)	DWKAFL-IDS	Improves convergence speed, improved prediction accuracy	Lower accuracy on some datasets (e.g., 85% on NSL-KDD), increased complexity, communication overhead in large-scale deployments.
Beuran (2025)	FedMSE	Achieves high detection accuracy (up to 97.30%), reduces learning costs by requiring only 50% gateway participation, and handles heterogeneous IoT data effectively	Requires careful hyperparameter tuning and efficient gateway selection, extra server-side computations can add complexity

## 3 Preliminaries

### 3.1 Tab transformer

The tab transformer Huang et al. (2020) is a novel transformer model developed specially for handling tabular data. It resulted from the success of transformer models in Natural Language Processing (NLP), vision, and other domains. The researchers, who developed the TTF, studied how transformer models can be used to incorporate features of the dataset and then generalize much better in more varied scenarios than usual models, by leveraging the inherent capabilities of the transformer models to capture dependencies among various features. Its design enables it to use not only categorical but also continuous features of a tabular dataset. TTF helps the users in automated feature engineering, whereas it supersedes the traditional models in terms of accuracy and performance. One of the main characteristics of TTF is its capability to capture complex, long-range dependencies between variables in the tabular data, which is often hard to handle for other ML models.

The tab transformer enforces a mathematical model through self-attention, whereby it maps the input features to a latent space. It has an embedding layer for categorical variables and a numerical continuous layer for continuous variables. From the perspective of the self-attention mechanism of the TTF, this can be mathematically expressed as:


(1) $$\begin{array}{l} Attention\left(Q,K,V\right)=softmax\left(\frac{QK^{T}}{\sqrt{d_{k}}}\right)V, \end{array}$$

Here, *Q*, *K*, and *V* represent the query, key, and value vectors, respectively, and *d*_*k*_ is the dimensionality of the key vectors. The softmax function ensures that the attention weights across the input positions sum to one. This attention mechanism authorizes the model to weigh the volume of diverse features when making predictions.

**Figure 1:**
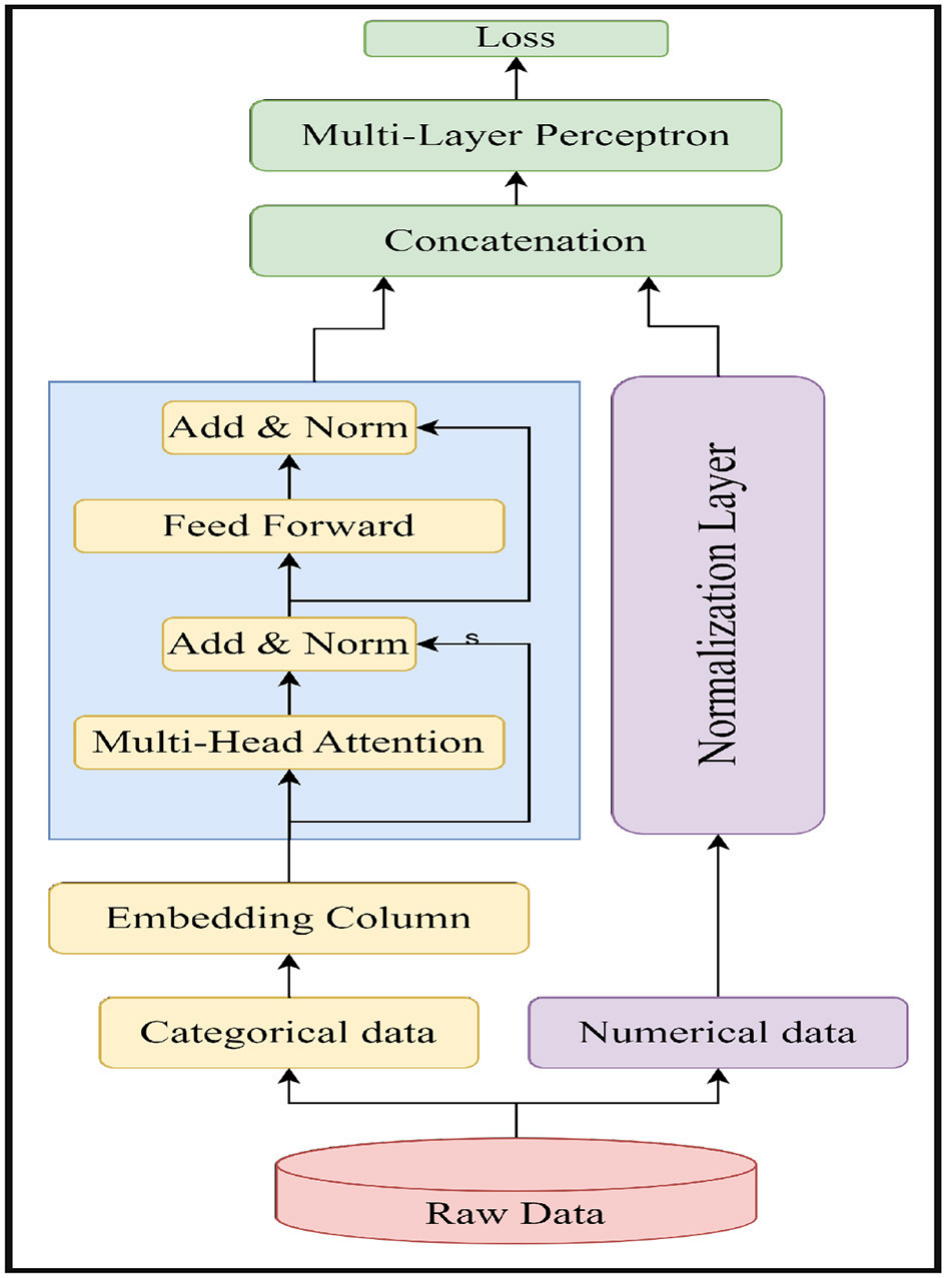
Flowchart of the tab transformer.

The overall flowchart of TTF is shown in Figure 1. The flowchart shows that the TTF first abstracts raw data inputs into two categorical and numerical streams. The categorical variables are then encoded into numerical embeddings, and the numerical data is standardized. Next, they proceed to multi-head attention to capture complex inter-feature relations, followed by residual connection and normalization for stability. The two processed data streams are then concatenated and fed into the MLP to further learn and abstract the data's features. After the output, it is compared with the true label, and the loss is then calculated to guide the optimizer through backpropagation for the training process. The architecture is designed to handle the complexity in tabular data in ML and evenly balance the unique characteristics of categorical and numerical data in a unified framework.

### 3.2 EEFO algorithm

Electric eels' group foraging behaviors, observed in nature (Zhao W. et al., 2023), inspire the EEFO (smart energy foraging optimization) algorithm. Mathematically, the electric eel foraging behaviors–interaction, resting, hunting, migration–are optimized for exploration and exploitation during the optimization procedure. An energy element is introduced to handle the shift from global search to local search and the harmony between exploration and exploitation in the search area.

- **Interaction:** The position update equation in the interaction behavior is given by:

(2) $$\begin{array}{l} X_{i}^{t+1}=X_{i}^{t}+\alpha_{i}^{t}\left(X_{j}^{t}-X_{i}^{t}\right) \end{array}$$

where $X_{i}^{t}$ and $X_{j}^{t}$ are the positions of the *i*-th and *j*-th eels at time *t*, respectively, and $\alpha_{i}^{t}$ is a number randomly selected in range [−1, 1].

- **Resting:** The energy update equation in the resting behavior is given by:

(3) $$\begin{array}{l} E_{i}^{t+1}=E_{i}^{t}+\beta_{i}^{t} \end{array}$$

where $E_{i}^{t}$ is the energy of the *i*-th eel at time *t*, and $\beta_{i}^{t}$ is a number randomly selected in range [0, 1].

- **Hunting:** The position update equation in the hunting behavior is given by:

(4) $$\begin{array}{l} X_{i}^{t+1}=X_{i}^{t}+\gamma_{i}^{t}\left(X_{best}^{t}-X_{i}^{t}\right) \end{array}$$

where $X_{best}^{t}$ is the position of the best eel at time *t*, and $\gamma_{i}^{t}$ is a number randomly selected in range [0, 1].

- **Migration:** The position update equation in the migration behavior is given by:

(5) $$\begin{array}{l} X_{i}^{t+1}=X_{i}^{t}+\delta_{i}^{t}L_{i}^{t} \end{array}$$

where $L_{i}^{t}$ is a Levy flight, and $\delta_{i}^{t}$ is a number randomly selected in range [−1, 1].

**Figure 2:**
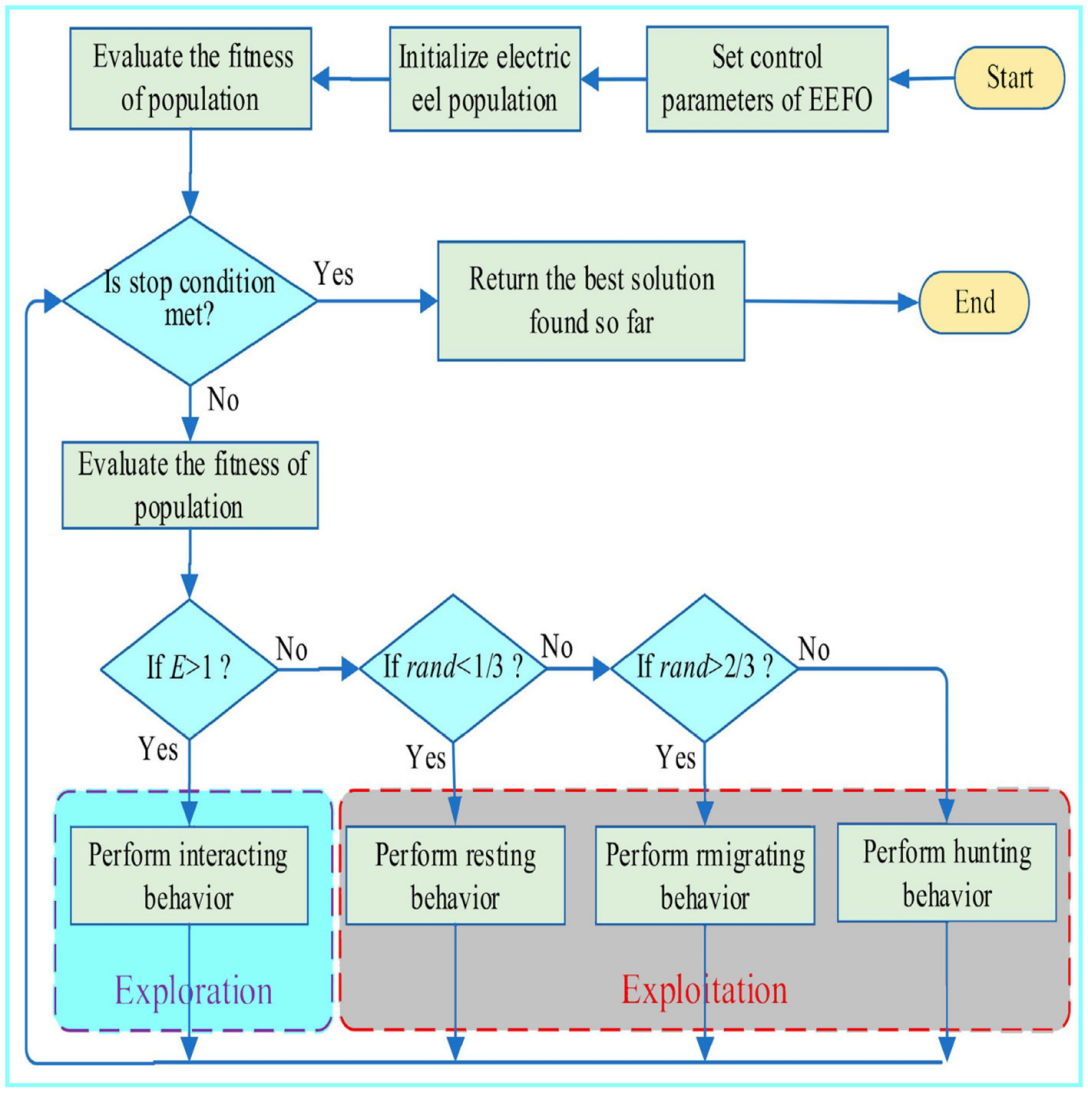
Flowchart of the EEFO optimizer.

An algorithm should ensure that the positions and energies of the eels are within their respective bounds. If a position or energy value goes beyond bounds, it should be reset to a reasonable value. It also needs to assess the fitness of each eel after updating its situation and update the best eel if necessary. The algorithm continues with these steps until a stopping condition is satisfied, for example, reaching a predetermined number of iterations or achieving a certain fitness level. The flowchart depicting EEFO is shown in Figure 2.

### 3.3 FL-based IDS frameworks utilizing deep learning models

The following subsection briefly introduces the general framework for malware detection in IoT environments, as proposed by Rey et al. (2022), emphasizing scenarios where data privacy and integrity are critical. The framework assumes a server and multiple clients owning data from different IoT devices. The server coordinates the FL process and aggregates the models sent by local clients. Local clients train their models using their respective datasets and transmit the updated model parameters to the server. The framework is evaluated using the N-BaIoT dataset, which contains data from nine IoT devices infected with either *Mirai* or *BASHLITE* malware. The study explores supervised and unsupervised approaches for malware detection, employing MLPs and autoencoders as the primary model architectures. The study also examines the impact of adversarial attacks on the FL process, where malicious clients may submit manipulated model updates to the server. It evaluates various aggregation functions as countermeasures to mitigate the effects of such attacks. The paper discusses the difficulties and limitations of the framework, for example, the communication and computation costs, the heterogeneity in the data, the model robustness, and the deployment in *B*5*G* scenarios.

**Figure 3:**
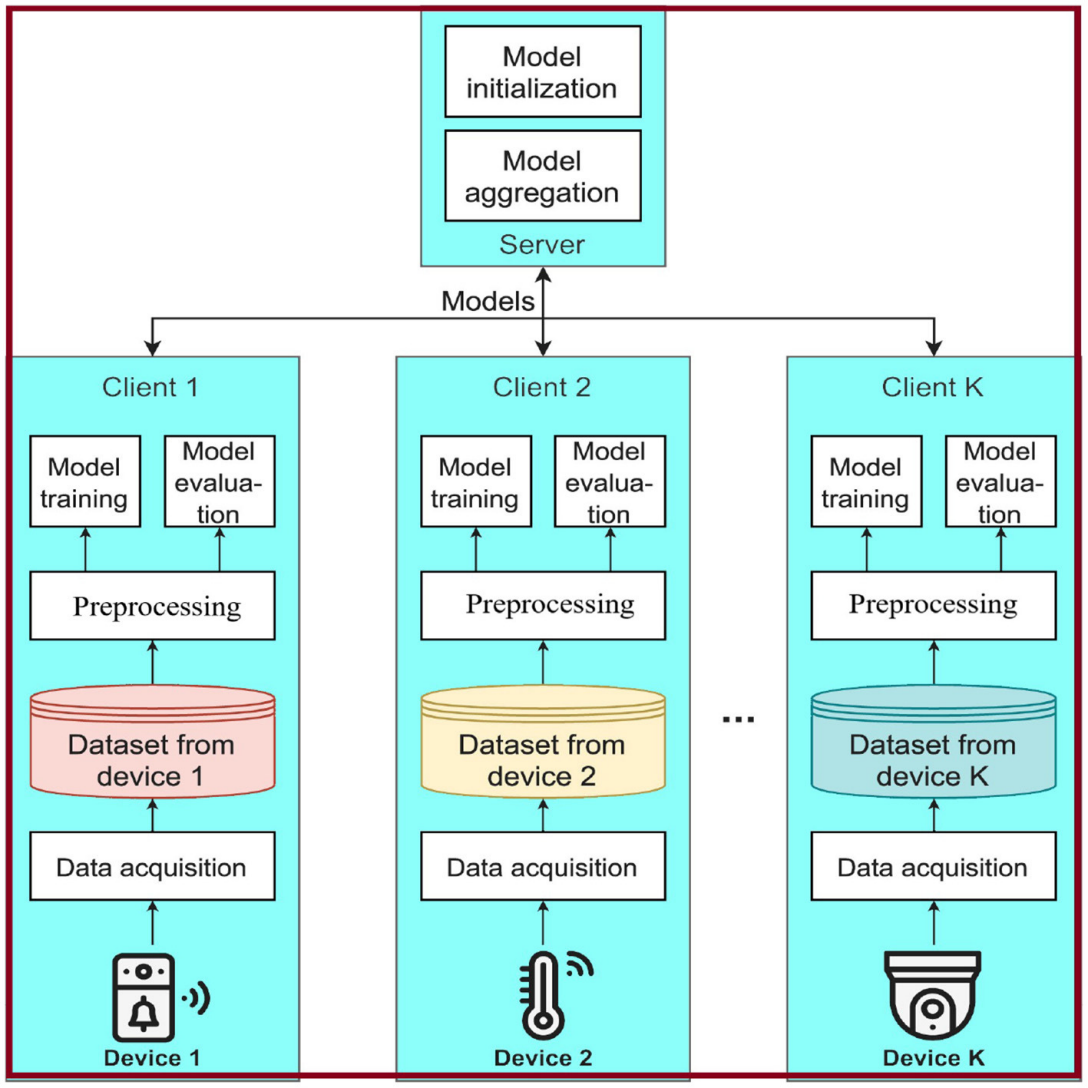
Framework of the FL-based IDS frameworks.

The framework, illustrated in Figure 3, compares the FL-based IDS framework with conventional methodologies, such as a naive decentralized model where clients perform local training and testing, a centralized model lacking privacy preservation, and two FL-based IDS framework variants utilizing Mini-batch and Multi-epoch aggregation algorithms. The objective is to determine the feasibility of using the federated approach in IoT malware detection scenarios by comparing the average performance of these models.

A key limitation of this framework is the small number of clients used for training, currently limited to eight, which is sufficient for the dataset employed but inadequate for larger-scale applications. In the real-world scenario of a 5*G* (Beyond 5*G*, or B5G) network, device deployments are expected to reach up to 10 million devices per square kilometer, as per the *ITU* (International Telecommunication Union) requirements (Series, 2021). Another challenge is hyperparameter selection in FL-based IDS frameworks as there are difficulties with non-IID data. Section 4 proposes solutions to address these challenges.

## 4 Proposed Method

The innovation of the proposed Framework can be outlined as using the TTF model for classification and the EEFO algorithm for hyperparameter optimization, integrated within the architectural framework (Rey et al., 2022). We replace the model used in the original framework, i.e., the neural network model and autoencoder (discussed in Section 3.3), with the TTF model. This integration of the TTF transformer with FL-based IDS frameworks is motivated by its ability to process data efficiently. Unlike the neural network and autoencoder models, the TTF effectively captures feature dependencies through self-attention mechanisms and enhances detection accuracy by reducing computational overhead in FL-based IDS environments. Additionally, we replace traditional grid search techniques with the EEFO algorithm. Unlike Grid Search, which exhaustively evaluates all hyperparameter combinations, EEFO uses an adaptive search strategy inspired by electric eels' foraging behavior, allowing it to explore the hyperparameter space efficiently. This replacement reduces computational overhead while optimal performance is increased in FL-based IDS frameworks, due to training resources being distributed across multiple clients.

The Subsection 3.3 has also identified some shortcomings within the FL-based IDS framework (Rey et al., 2022), particularly the constraint of a limited number of clients interacting with the model. The TTF model can improve performance when handling sparse data distributed across clients. Unlike the neural network model used in the framework, which is constrained by the number of clients, the TTF model is designed to process and learn more effectively from the heterogeneous data generated by IoT devices. Unlike the neural network model used in this framework, with the limitation in the number of clients, TTF is capable of handling large-scale, high-dimensional tabular data, which can enhance the generalization of the system across diverse environments in IoT.

Furthermore, we developed an approach combining grid search and EEFO to address certain limitations of hyperparameter tuning. This optimization technique is designed to streamline hyperparameter selection, significantly reducing the computational load and ensuring optimal performance in a shorter period. The use of EEFO would further help to enhance the precision of FL-based IDS frameworks so that robust IDSs are realized even with a higher number of deployments that shall cater to the ITU standards for B5G networks. Since architectural hyperparameters must be consistent across clients, they require consensus through shared validation results. The EEFO optimization algorithm facilitates this process. Federated clients transmit the verification findings for each possible combination of hyperparameters, and the EEFO algorithm identifies the optimal solution based on the average performance across all clients. This ensures that hyperparameter selection is driven by achieving the highest validation accuracy.

Our tuning process for each client includes the learning rate, number of epochs, and the batch size. The values of these parameters were generated randomly by the following Equation 6:


(6) $$\begin{array}{l} X=L+\text{rand}\times \left(U-L\right),\;L=\left[0.0001,5,8\right],\;U=\left[0.1,50,128\right] \end{array}$$

where *L* and *U* are the lower and upper bounds for the hyperparameters, respectively. *rand* is a random number between 0 and 1. This equation linearly interpolates between the lower and upper bounds based on the random number. It effectively samples from a uniform distribution within these bounds. For instance, learning rates were explored between 0.0001 and 0.1, the number of epochs ranged from 5 to 50, and batch sizes varied from 8 to 128.

**Figure 4:**
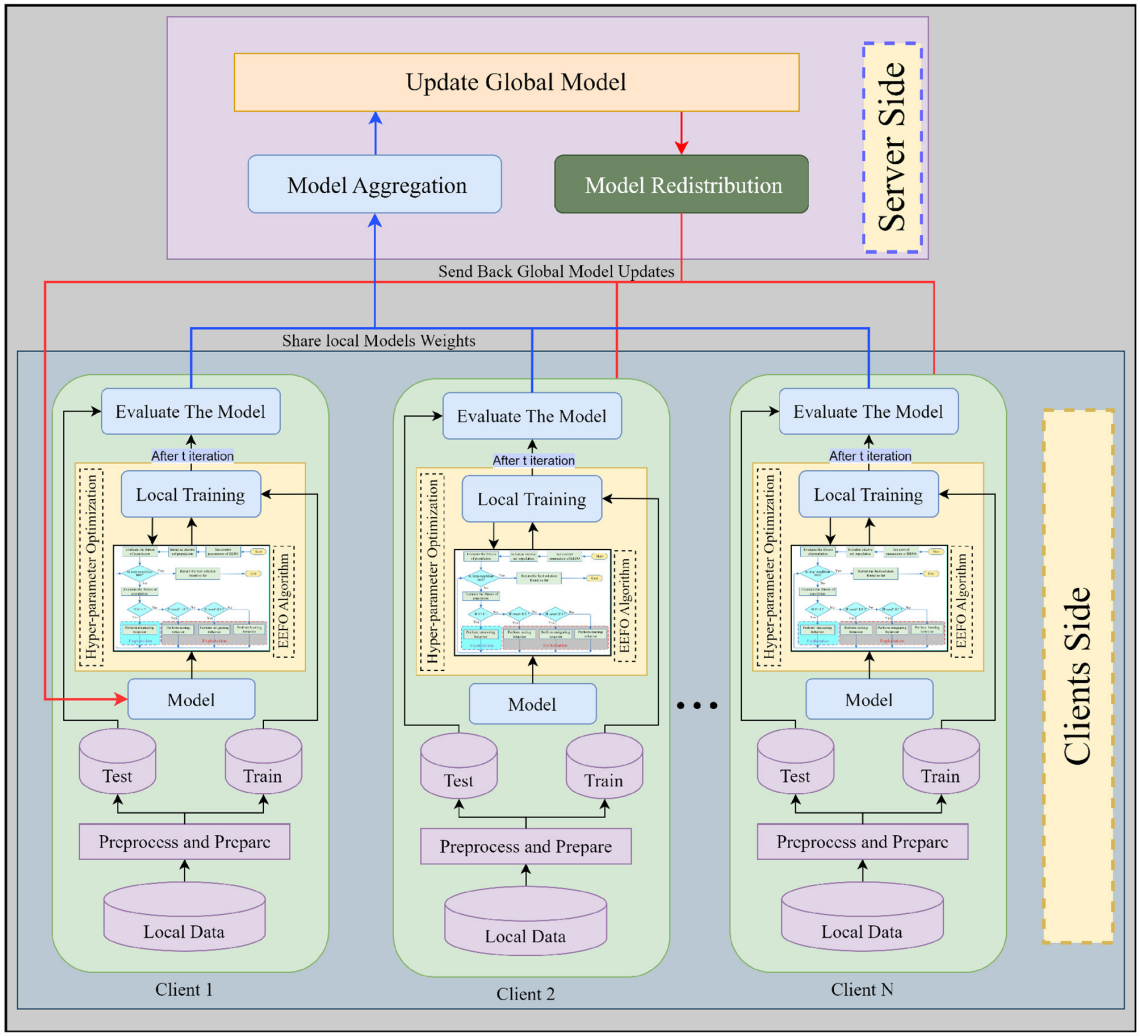
Flowchart of the proposed framework.

All participants must agree upon architectural hyperparameters based on shared validation results. The validation is carried out using benign data to minimize loss. Hyperparameter selection is based on achieving the highest validation accuracy. Figure 4 presents the proposed FL-based IDS framework based on TTF and EEFO algorithms as hyperparameter optimization. Where all clients collaboratively contribute to the development of a shared global model (Weights) under the coordination of a central server. The process begins at the client level, where each client preprocesses and prepares its local data, optimizes the hyperparameters, and trains the model locally using this data. These locally trained models' weights are then shared with the central server. At the server side, the model weights received from all clients are aggregated to update the global model. This aggregation aims to synthesize the learning from diverse data sources and improve the overall model by incorporating a broad spectrum of data characteristics and patterns. Once the global model is updated, it is returned to all clients.

This cyclical process of training, sharing, aggregating, and redistributing enhances the model iteratively while maintaining data privacy, as the data remains local to each client, preventing privacy breaches and data leakage. The proposed framework could be summarized as follows stages:

- **Stage 1: Dataset Loading and Preparation** The loaded dataset is preprocessed and divided into training and testing sets at this stage. The training part was utilized to train and tune the model parameters. Then, after *t* iterations, the testing part is used to evaluate the model at the client.
- **Stage 2: Hyperparameter Optimization and Training** This stage performs hyperparameter tuning and training for the local model process for *t* iterations. At each iteration, the following two sub-stages were implemented:

- *Tuning Process By EEFO algorithm:* In conventional FL frameworks, hyperparameters remain unchanged across all clients, disregarding that individual clients may have varying amounts of data and computational resources. By using the EEFO algorithm, each client optimizes its model's hyperparameters such as learning rate, number of epochs, and batch size, based on Equation 6, on the local data. This optimization ensures that the model is tuned to the particular characteristics of the data it trains on. Each client's model is specifically tailored to the unique characteristics of its own data and scenario, for instance, in IoT environments where data properties can vary significantly from one device to another due to differences in device types, configurations, or operational scenarios. This tuning process ensures that the model is accurate and efficient in learning from the specific data it encounters. The stage enhances the overall FL framework process by ensuring that when local models are aggregated, each has been optimized under the best possible conditions for its dataset and according to its resources.
- *Local Model Training:* Each client trains its pre-optimized model using its local training data. The client then revisits the hyperparameter optimization process. This iterative process benefits from the initial hyperparameter optimization, enabling a more accurate and effective learning phase.

- **Stage 3: Local Model Evaluation** At this stage, the model is evaluated using the testing part of the dataset resulting from stage 1. This stage is important for assessing the local model performance. The utilized evaluation metrics are discussed later in Section 5.2.
- **Stage 4: Sharing Model Weights** After the fulfillment of training and evaluation, the model at each client shares its parameter weights to the central server. The server combines these weights to assemble an updated global model representing the summed learning across all the clients.
- **Stage 5: Model Redistribution and Iteration** The enhanced global model is redistributed to all local clients for repeat training at this stage. This cycle of training, optimizing, sharing, and updating continues until reaching a predetermined termination criterion. This ensures that the model is continuously improving and adaptable to new data or threats.

## 5 Experiments and results

In our experiments, each client processes its dataset independently, adhering to FL principles, thereby ensuring decentralized data handling within the framework. The strategy emphasizes data privacy and scalability over the distributed networks. Finally, our approach focuses exclusively on supervised learning, utilizing labeled data for both binary and multi-class classification tasks. This strategy was implemented to train the TTF model, enabling successful intrusion detection in IoT devices.

In the proposed framework, the initial TTF model with randomly initialized parameters serves as the foundational model, which is then fine-tuned using the EEFO algorithm. Afterward, this optimized model is dispatched by the server to the clients for multiple iterations of local training. The primary evaluation focuses on the implementation improvements of the proposed framework, particularly in comparison to the FedAvg algorithm (McMahan et al., 2017), using metrics such as client workload, time to convergence, and accuracy. To quantify the impact of network latency, the proposed framework was implemented in a single computational environment. In this approach, a unified Python process handles both server and client roles on the same hardware, utilizing PyTorch 23.05 to facilitate communication between local processes. It should be clarified that this methodological decision does not alter the overall applicability or relevance of our findings.

This subsection summarizes the datasets used and presents the results of various experiments conducted to validate the proposed framework. All experiments took place using a *DELL* laptop equipped with a *corei*7 processor, 32*GB* of *RAM*, and powered by an Nvidia GTX 1650*TI* graphics card.

### 5.1 Datasets details

To ensure the performance of the proposed model, we conduct the experiments on three different datasets, which are summarized in the following subsections.

- **N-BaIoT dataset:** The N-BaIoT dataset offers real data from Mirai and BASHLITE botnet-infected IoT devices (Meidan et al., 2018). The dataset contains data from *nine* retail IoT devices that were certified contaminated by Mirai and BASHLITE. This dataset discloses the deficiency of shared botnet datasets primarily for the IoT, and provides real data for research purposes. This makes it valuable for research and development in the specialization of IoT botnet attack detection. N-BaIoT is valuable for IDS and multi-class classification applications, despite its relatively limited size and potential class imbalance. Table 2 summarizes the description of this dataset.
- **UNSW-NB15 dataset:** We evaluate the model using the UNSW-NB15 dataset (Moustafa and Slay, 2015), which simulates various contemporary attack types in a current network setting. We used this dataset in a binary layout, where each data sampling is assigned either a Benign or an Attack label. The actual dataset has approximately 2.5 million records, encompassing a total of 48 features, as documented in Table 3. To use this dataset in a federated setting, we assumed that every unique IP address denotes a distinct participant. In this way, the data instances are dispersed and established on the goal IP address present within the network packets. We identified those participants whose local datasets contained at least one attack sample during the selection process. We identified 10 distinct nodes that met these criteria. Table 4 summarizes the samples associated with these 10 participants.
- **CICIoT2023 Dataset:** The CICIoT2023 dataset is a real-time dataset for extensive-scale incursions in an IoT (Neto et al., 2023). It is a comprehensive IoT invasion dataset proposed to enhance the expansion of security analysis applications in real IoT procedures. The dataset consists of 33 incursions executed in an IoT network of 105 instruments with 46 features. These incursions are categorized into 7 classes: DoS, DDoS, Web-based, Recon, Spoofing, Mirai, and Brute Force. Each attack is performed by malicious IoT devices on different IoT instruments. Table 5 provides a general dataset description.

**Table 2:** N-BaIoT dataset descriptions.

**Property**	**Value**
Number of features	89
Number of samples	7,062,606
Number of classes	11 (10 attack classes + 1 benign class)

**Table 3:** UNSW-NB15 dataset descriptions.

**Property**	**Value**
Number of features	48
Number of samples	2,500,000
Number of classes	9 (Fuzzers, Analysis, Backdoors, DoS, Exploits, Generic,
	Reconnaissance, Shellcode, Worms)

**Table 4:** Selected distributed UNSW-NB15 dataset for local training.

**Local dataset**	**Benign samples**	**Attack samples**	**Total**
1	38,082	2,911	40,993
2	8,231	5,008	13,239
3	25,812	2,497	28,309
4	11,719	4,826	16,545
5	39,007	3,429	42,436
6	43,754	3,741	47,495
7	6,931	4,609	11,540
8	21,080	3,991	25,071
9	92,991	2,671	95,662
10	11,195	3,839	15,034

**Table 5:** CICIoT2023 dataset descriptions.

**Property**	**Value**
Number of features	46
Number of samples	46,686,579
Number of classes	34 (33 attack classes + 1 benign class)

In our research, we considered the composition of the datasets in this respect, ensuring they are balanced across classes. For the datasets used in this study–N-BaloT, UNSW-NB15, and CICIoT2023–measures were taken to ensure equal distribution of samples between classes. This helps mitigate biases and helps a model to showcase its generalizing capabilities fairly across various scenarios within IoT security frameworks. By ensuring dataset balance, the research findings are reinforced, providing a reliable basis for assessing the performance of the proposed FL framework.

### 5.2 Evaluation metrics

Accuracy is a measure that indicates the ratio of valid forecasts assembled by a model in association with all predictions. It is derived from the summation of true positive (TP) and true negative (TN) predictions divided by all predictions that are false positive (FP) and false negative (FN). Mathematically, accuracy is calculated as:


(7) $$\begin{array}{l} \text{Accuracy}=\frac{\text{TP}+\text{TN}}{\text{TP}+\text{TN}+\text{FP}+\text{FN}} \end{array}$$

Precision is one performance gauge, quantifying the ratio of correctly identified positive predictions against all positive predictions assembled by the model. It is obtained by the division of true positive (TP) predictions by the full of true positive and false positive (FP) predictions. Mathematically, precision is represented as:


(8) $$\begin{array}{l} \text{Precision}=\frac{\text{TP}}{\text{TP}+\text{FP}} \end{array}$$

Recall, also directed to as sensitiveness or true positive rate, signifies the ratio of correctly identified actual positive cases by the model relative to the total number of actual positive cases. It's computed as TP divided by (TP + FN). Mathematically, recall is expressed as:


(9) $$\begin{array}{l} \text{Recall}=\frac{\text{TP}}{\text{TP}+\text{FN}} \end{array}$$

The F1 score is a balanced metric that integrates both precision and recall in an overall performance rating of a model. It is calculated as the harmonic mean of the Precision and Recall terms, hence it considers both the model's true positive rate (Recall) and positive predictive value (Precision). Mathematically, the F1 score can be defined as:


(10) $$\begin{array}{l} \text{F1-Score}=2\cdot \frac{\text{Precision}\cdot \text{Recall}}{\text{Precision}+\text{Recall}} \end{array}$$

One of the most important assessments essential in measuring the performance of the IDS model in the IoT is the stated metrics. For example, the accuracy of the model generally provides an overall view of how well the model performs. Precision and recall, on the other hand, disclose how well the model can identify positive cases (intrusions) and prevent false alarms. Moreover, minimizing the false positive rate is significant since it reduces the number of false alarms while preserving the credibility of the model.

### 5.3 Comparative results

The evaluation of our proposed framework is based on the N-BaIoT, UNSW-NB15, and CIC-IoT2023 datasets (explained in Section 5.1). Table 6 presents performance results accuracy, loss, and processing time (in s) of the training process for the Proposed & Original framework over the three datasets in both Binary & Multi classification tasks. The table also presents a comparison according to computational cost (time in s) between the proposed and the original framework. It is noted that the usage of the EEFO algorithm as hyperparameter optimization reduced the time needed during the training phase. From this table, the proposed model achieved the highest accuracy, lowest loss, and less time over the original.

**Table 6:** Training performance results of binary and multiclass classification for original and proposed frameworks.

**Dataset**	**Framework**	**Binary**			**Multi-class**		
		**Accuracy (%)**	**Loss**	**Time(s)**	**Accuracy (%)**	**Loss**	**Time(s)**
N-BaIoT	Original	97.1	0.28	520	96.00	0.32	680
	Proposed	**99.95**	**0.02**	**400**	**99.05**	**0.08**	**550**
UNSW-NB15	Original	96.50	0.25	610	94.92	0.34	820
	Proposed	**99.01**	**0.05**	**480**	**98.30**	**0.12**	**650**
CICIoT2023	Original	95.00	0.32	920	93.54	0.37	1250
	Proposed	**99.94**	**0.03**	**750**	**98.65**	**0.12**	**1020**

The bold values indicate that these are best values compared to others.

**Figure 5:**
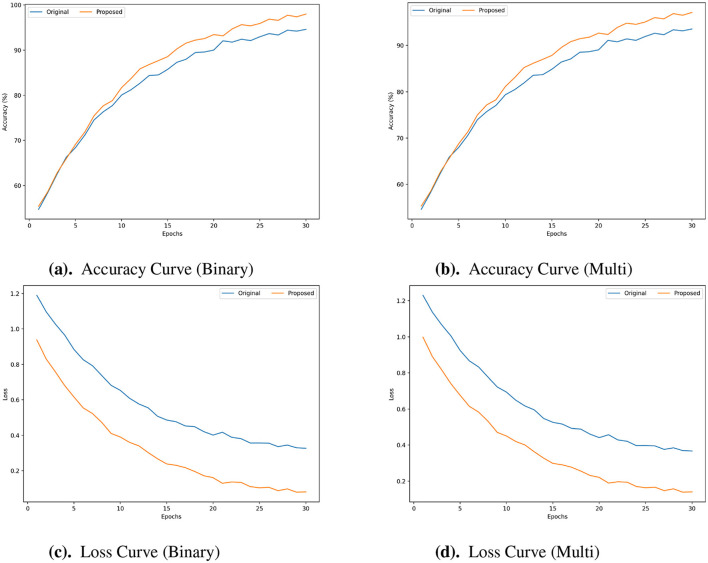
**(a–d)** Accuracy and loss curves for proposed vs. original framework in both binary and multi-class for N-BaIoT datasets.

**Figure 6:**
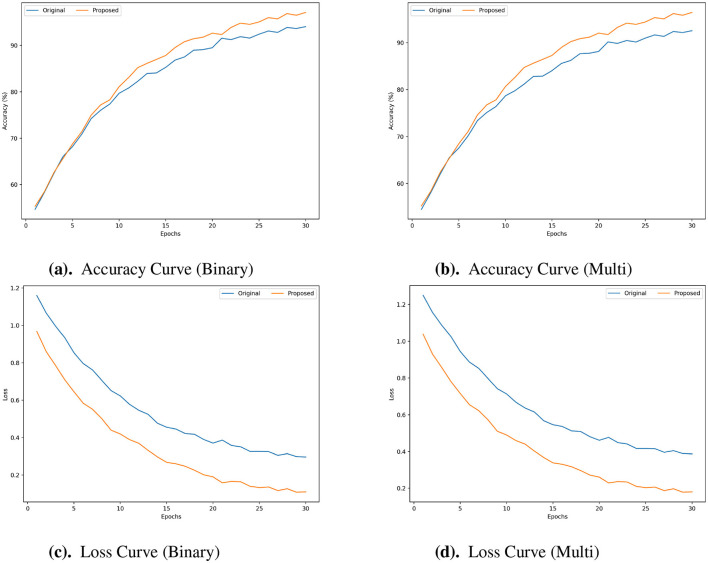
**(a–d)** Accuracy and loss curves for proposed vs. original framework in both binary and multi-class for UNSW-NB15 datasets.

**Figure 7:**
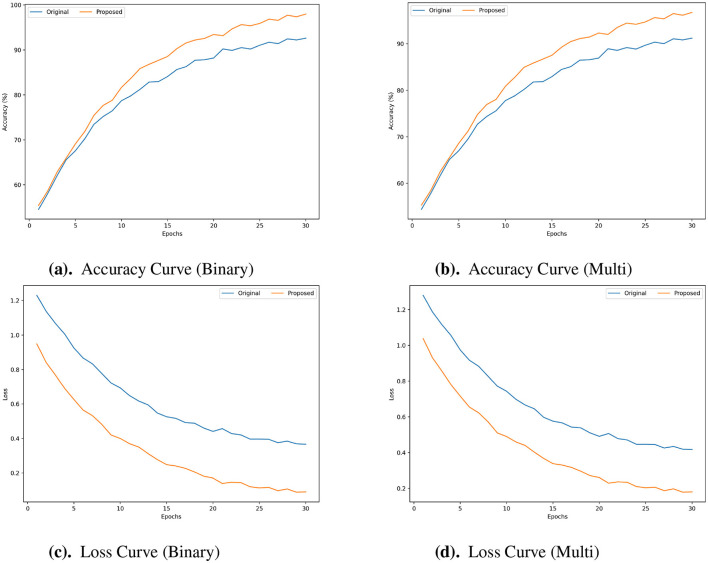
**(a–d)** Accuracy and loss curves for proposed vs. original framework in both binary and multi-class for CICIoT2023 datasets.

The accuracy curves presented in Figures 5a, b, 6a, b, 7a, b are evident that the proposed framework achieved higher accuracy across the epochs for both classification tasks. These curves show improved accuracy levels and faster convergence. In terms of efficiency and model generalization, the proposed framework outperforms the original framework since it reaches peak accuracy earlier and has a steeper improvement trajectory. The advantages of the proposed framework are further shown by the Loss curves (Figures 5c, d, 6c, d, 7c, d). When compared to the original framework, the proposed framework exhibits a much quicker decrease in loss during training and maintains a lower loss value across all epochs. This reduced loss, which is seen in both the binary and multi-class cases, shows improved optimization capabilities and more efficient learning with fewer training mistakes. The accuracy & loss curves presented in Figures 5–7 illustrate the superior performance of the proposed framework compared to the Original for both Binary and Multi Classification tasks over the N-BaloT, UNSW-NB15, and CICIoT2023 datasets, respectively.

The performance results of our model in the testing phase are presented in Table 7 in the binary classification case over the three datasets. For the N-BaloT dataset, the proposed framework achieves the highest values in accuracy of 99.92%, precision of 99.80%, recall of 99.82%, and an F1-score of 99.81%, exceeding the original's metrics of 97.83%, 97.70%, 97.60%, and 97.65%, respectively. Similarly, on the UNSW-NB15 dataset, the proposed framework achieved an accuracy of 98.50%, precision of 98.30%, recall of 98.20%, and an F1-score of 98.25%, compared to the original framework's 96.48%, 96.30%, 96.10%, and 96.20%. The majority of the improvements are observed in the CICIoT2023 dataset, where our Proposed framework achieves 99.86% accuracy, 99.75% precision, 99.55% recall, and 99.65% F1-score, significantly higher than the original framework's 95.38%, 95.25%, 95.05%, and 95.10%. Additionally, the proposed framework requires less time during the testing phase than the original. These results indicate that our proposed framework consistently outperforms the original framework across all three datasets in binary classification tasks.

**Table 7:** Testing performance results of the proposed and original frameworks in binary classification.

**Dataset**	**Framework**	**Accuracy (%)**	**Precision (%)**	**Recall (%)**	**F1 (%)**	**Time (s)**
N-BaIoT	Original	97.83	97.70	97.60	97.65	210
	Proposed	**99.92**	**99.80**	**99.82**	**99.81**	**160**
UNSW-NB15	Original	96.48	96.30	96.10	96.20	275
	Proposed	**98.50**	**98.30**	**98.20**	**98.25**	**210**
CICIoT2023	Original	95.38	95.25	95.05	95.10	430
	Proposed	**99.86**	**99.75**	**99.55**	**99.65**	**350**

The bold values indicate that these are best values compared to others.

In addition, the performance results of our model in the testing phase are shown in Table 8 in the Multi classification case over the three datasets. For the N-BaloT dataset, the proposed framework achieves an accuracy of 99.20%, precision of 99.00%, recall of 98.90%, and an F1-score of 98.95%, outperforming the original framework's metrics of 96.50%, 96.00%, 95.80%, and 95.90%, respectively. Similarly, on the UNSW-NB15 dataset, the proposed framework records an accuracy of 98.04%, precision of 97.95%, recall of 97.83%, and an F1-score of 97.89%, when compared to the original's 94.50%, 94.20%, 94.00%, and 94.10%. Finally, for the CICIoT2023 dataset, the proposed framework achieves 98.80% accuracy, 98.60% precision, 98.50% recall, and 98.55% F1-score, surpassing the original framework's 93.00%, 92.80%, 92.50%, and 92.65%. Also, the proposed processing time is consistently less than the original. These results prove that our proposed framework always outperforms the original framework over the utilized datasets in the Binary & the mutli-classification tasks.

**Table 8:** Testing performance results of the proposed and original frameworks in multiclass classification.

**Dataset**	**Framework**	**Accuracy (%)**	**Precision (%)**	**Recall (%)**	**F1 (%)**	**Time (s)**
N-BaIoT	Original	96.50	96.00	95.80	95.90	320
	Proposed	**99.20**	**99.00**	**98.90**	**98.95**	**250**
UNSW-NB15	Original	94.50	94.20	94.00	94.10	430
	Proposed	**98.04**	**97.95**	**97.83**	**97.89**	**350**
CICIoT2023	Original	93.00	92.80	92.50	92.65	620
	Proposed	**98.80**	**98.60**	**98.50**	**98.55**	**500**

The bold values indicate that these are best values compared to others.

### 5.4 Comparative with state-of-the-art methods

The comparative analysis in this section characterizes recent FL-based solutions in the domain of IDS, which is given in Table 9. Models used FL frameworks under comparison span Deep Neural Networks (DNN), LSTM, Random Forest (RF), as well as specialized frameworks like FLUIDS (Aouedi et al., 2022) and Energy Flow Classifier (EFC) (Carvalho Bertoli et al., 2023) along with SIDS (Amiri-Zarandi, 2023) and FL-IIDS (Jin et al., 2024). Research studies cite a spectrum of datasets, including variations of UNSW-NB15, KDD99, NSL-KDD, CIC-IDS-2017, Bot-IoT, TON-IoT, and CSE-CIC-IDS-2018, which present unique challenges representative of real-world IoT security scenarios. Recently, Olanrewaju-George and Pranggono (2025) introduced the unsupervised AutoEncoder and supervised DNN applied on N-BaIoT dataset, they achieved 90.93% of accuracy. They achieved 93.12% of F-score values, which are low compared to the proposed. The DWKAFL-IDS model (Wen et al., 2025) is tested on CICIDS2017, UNSW-NB15, and NSL-KDD datasets, and it achieved 91.38% of accuracy on the UNSW-NB15 dataset. Furthermore, the FedMSE model (Beuran, 2025) evaluated on the N-BaIoT dataset and achieved 94.74% of accuracy. It is noted from the literature summarized in the Table 9 that the proposed model shows outstanding performance with high accuracy and F-score values across the tested datasets. On the N-BaIoT dataset, the model averages accuracy and F-score values of 99.02% and 98.87%, respectively, clearly indicating superior detection capabilities compared to other models on the same dataset.

**Table 9:** Comparison with recent FL-based frameworks.

**References**	**Model-based**	**Dataset**	**Accuracy (%)**	**F-Score (%)**
Sarhan et al. (2023)	DNN	NF-UNSW-NB15-v2 (Sarhan et al., 2022)	91.16	90.51
	LSTM		88.92	88.38
	DNN	NF-BoT-IoT-v2 (Sarhan et al., 2022)	93.08	93.01
	LSTM		92.57	92.52
Markovic et al. (2022)	RF	KDD99	84.77	-
		NSL-KDD	93.51	-
		UNSW-NB15	79.13	-
		CIC-IDS-2017 (Sharafaldin et al., 2018)	73.26	-
Aouedi et al. (2022)	FLUIDS	UNSW-NB15	-	86
Carvalho Bertoli et al. (2023)	EFC	Bot-IoT	93	96
		TON-IoT (Moustafa, 2021)	74	77
		UNSW-NB15	97	73
		CSE-CIC-IDS-2018 (Gopalan, 2021)	98	90
Amiri-Zarandi (2023)	SIDS	UNSW-NB15	84	90
Jin et al. (2024)	FL-IIDS	UNSW-NB15	68.764	-
		CSE-CIC-IDS-2018	99.62	-
Olanrewaju-George and Pranggono (2025)	AutoEncoder and DNN	N-BaIoT	90.93	93.12
Wen et al. (2025)	DWKAFL-IDS	UNSW-NB15	91.38	-
Beuran (2025)	FedMSE	N-BaIoT	94.74	-
Proposed		N-BaIoT	**99.02**	**98.87**
		UNSW-NB15	UNSW-NB15	**98.02**
		CICIoT2023	**95.47**	**95.65**

Additionally, the model's implementation on the UNSW-NB15 and CICIoT2023 datasets validates its position, outperforming the results of other contemporary FL-based solutions. In summary, Table 9 highlights the proposed model as a strong contender among FL-based IDS frameworks for IoT security, demonstrating its potential to emerge as a leading methodology based on the results presented.

### 5.5 Key findings and limitations

The presented results significantly improve ID in IoT environments compared to traditional FL-based IDS approaches. The results showed that our model achieved higher accuracy, precision, recall, and F1-score across multiple datasets (N-BaIoT, UNSW-NB15, and CICIoT2023). The EEFO algorithm enabled adaptive hyperparameter tuning and optimizing model performance based on dataset variations across different clients. Also, the TTF enhances feature representation, which leads to improved classification performance for both binary and mutli-classification ID tasks. The experimental results confirm that our approach outperforms existing FL-based IDS frameworks by achieving higher detection rates while maintaining data privacy and reducing central processing overhead.

Despite these advantages, the proposed framework has certain limitations. First, the computational costs of training the TTF on edge devices are significantly high, especially on the constrained resources of IoT environments. While FL-based IDS frameworks reduce the need for centralized data computation, local training on devices with constrained computational resources can introduce latency. In addition, the optimization process using EEFO requires iterative hyperparameter tuning, possibly increasing convergence time under situations of a huge number of clients. The second challenge is managing extremely unbalanced datasets, especially where specific attack types are underrepresented, possibly compromising the overall generalization of the model in real-world deployment.

## 6 Conclusion and future studies

Based on the in-depth analysis conducted in this study, we conclude that the proposed framework is highly significant for intrusion detection (ID) in IoT environments. The framework was evaluated on three diverse datasets–N-BaIoT, UNSW-NB15, and CIC-IoT2023–and demonstrated superior performance to traditional FL-based IDS frameworks. The tab transformer (TTF) model has proven highly effective in managing high-dimensional data and leveraging the intrinsic patterns inherent in IoT devices. By employing the electric eel foraging optimization (EEFO) algorithm for hyperparameter optimization, our model achieves high accuracy while maintaining resilience against various types of intrusions. The proposed framework exemplifies the potential of integrating advanced TTF architectures with nature-inspired optimization algorithms within a Federated Learning (FL) approach for intrusion detection systems (IDS). This approach can drive significant advancements in securing IoT devices.

As a future study, it would be valuable to explore the effects of unsupervised learning contexts to assess their impact in comparison to the supervised framework. Additionally, evaluating the robustness of the model in detecting attacks–particularly by testing it against manipulated adversarial samples designed to evade detection–presents a promising avenue for future research.

## Data Availability

Publicly available datasets were analyzed in this study. This data can be found here: https://www.kaggle.com/datasets/mkashifn/nbaiot-dataset, https://research.unsw.edu.au/projects/unsw-nb15-dataset, https://www.unb.ca/cic/datasets/iotdataset-2023.html.
